# Analytical and Diagnostic Performance of a Dual‐Target Blood Detection Test for Hepatocellular Carcinoma

**DOI:** 10.1002/cnr2.70017

**Published:** 2024-09-26

**Authors:** Qiankun Yang, Lanlan Dong, Lianglu Zhang, Wei Zhang, Yan Zhang, Yue Huang, Huifang Jin, Hao Yang, Xing Liu, Yanteng Zhao

**Affiliations:** ^1^ Department of Transfusion The First Affiliated Hospital of Zhengzhou University Zhengzhou China; ^2^ Academic Department Wuhan Ammunition Life‐Tech Company, Ltd. Wuhan China; ^3^ Department of Infectious Disease and Liver Disease, the Second Hospital of Nanjing Affiliated to Nanjing University of Chinese Medicine Nanjing China

**Keywords:** cell‐free DNA, cut‐off value, hepatocellular carcinoma, limit of detection, methylation, precision

## Abstract

**Background:**

Surveillance approaches with high sensitivity and specificity for hepatocellular carcinoma (HCC) are still urgently needed. Previous studies have shown that methylation of GNB4 and Riplet can effectively diagnose HCC.

**Aims:**

This study plan to analyze the performance of a blood test for detecting HCC using GNB4 and Riplet methylation.

**Methods and Results:**

This study mainly investigated the analytical performance of the dual‐target HCC blood test (DT‐HBT), including cut‐off value, limit of detection (LOD), precision, analytical specificity, and coincidence rate. In addition, the detection performance for HCC was validated in 1030 clinical plasma samples (214 HCC and 816 non‐HCC). Plasma samples from 25 HCC patients after hepatectomy were collected to assess the feasibility of the kit for postoperative recurrence monitoring. All analytical performance of the DT‐HBT met prespecified requirements. The LOD for GNB4, Riplet, and β‐actin was 1% methylation/100 copies/μL with cut‐offs of 43, 43, and 35, respectively. The DT‐HBT showed excellent precision, within 5% CV. It had a specificity of 91.5% for detecting other cancers, and 100% for breast, lung, and bladder cancer. No cross‐reactions were observed with 9 potential interfering substances. The DT‐HBT achieved a 100% coincidence rate in detecting reference and clinical samples. The clinical performance study found that the kit showed a sensitivity of 81.7% for stage I HCC, and an overall sensitivity and specificity of 87.4% and 92.3%, respectively. The detection sensitivity for postoperative recurrent patients was 95.8%, with a specificity of 100%.

**Conclusion:**

The analytical performance of the DT‐HBT met prespecified criteria. It provided HCC patients with a reliable and high‐performing new blood test for the HCC diagnosis and surveillance.

**Trial Registration:**

ClinicalTrials.gov identifier: NCT05685524

## 1 Introduction

In 2022, there were 367 657 new cases of liver cancer in China, accounting for 42.4% of the global total, with 316 544 deaths, accounting for 41.7% of the global total. Approximately half of the global liver cancer deaths occurred in China [1, 2]. Hepatocellular carcinoma (HCC) is one of the main types of liver cancer, with high malignancy, insidious onset, rapid development [3]. Approximately 64% of HCC patients are initially diagnosed at the intermediate and advanced stage [4]. The 5‐year survival rate of HCC in China is only 14.1% [5]. Therefore, early diagnosis and intervention could improve the overall survival rate of HCC.

The guideline for HCC screening recommends HCC examination every 6 months for high‐risk populations in China. In addition to the recommended ultrasound combined with AFP, new blood molecular marker detection methods such as cell‐free DNA (cfDNA) methylation have also been proposed [6]. Cell‐free DNA (cfDNA) refers to DNA fragments with lengths of 150–200 bp released into the blood during necrosis or apoptosis of cells, and cfDNA shed from tumor cells is called circulating tumor DNA (ctDNA) [7]. CtDNA carries the genetic and epigenetic alterations of tumor cells, including mutations, copy number variations, chromosomal rearrangements, and methylation modifications [8, 9, 10, 11]. A large number of studies have shown that compared with traditional cancer detection methods, ctDNA methylation detection is safe and can achieve ultra‐early detection, precise treatment, and monitoring of tumor recurrence [11]. It is considered to be a promising alternative method for detecting various cancers, with extremely high diagnostic accuracy. CfDNA can aid imaging examinations and significantly improve the diagnostic performance for various clinical types of HCC, including small HCC < 2 cm [6].

Hepatectomy is the preferred radical treatment for HCC patients with CNLC stage Ia, Ib, and IIa, and is the major strategy for long‐term survival of HCC patients [3]. A lot of clinical studies have shown that the 5‐year recurrence rate after hepatectomy is approximately 70% [12], and the 5‐year recurrence rate for small HCC (≤ 2 cm) is between 50% and 60% [13]. The high recurrence rate severely affected the 5‐year survival rate of HCC patients. For operable HCC, recurrence and metastasis are the main factors for poor prognosis, and also the leading causes of death [14, 15]. Therefore, early diagnosis of HCC recurrence and individualized treatment have become the focus of improving the survival of HCC patients. Currently, the clinical monitoring of HCC metastasis and recurrence mainly relies on imaging examinations and detection of traditional tumor markers, making it rather difficult to discover metastasis or recurrence at an early stage [16]. Therefore, the search for new biomarkers to predict early recurrence of HCC is of great significance for prolonging the postoperative survival of HCC patients.

Most of the currently marketed blood‐based cancer detection products use cfDNA methylation markers, with fluorescence PCR as the detection technology, such as RNF180/Septin9 gene methylation for gastric cancer detection, Septin9 gene methylation for colorectal cancer detection, and SHOX2/RASSF1A/PTGER4 gene methylation for lung cancer detection [17, 18, 19]. Fluorescent PCR was considered to be a cost‐effective assay, so detecting cfDNA with this method is economically advantageous for extensive clinical application [20]. Among the HCC blood detection kits that have obtained China Class III medical device registration certificate, there are only two. One was a combination of 7 miRNAs [21], and the other conducted DNA methylation detection with ddPCR technology [22]. Both kits were intended for auxiliary diagnosis of HCC, but there were no relevant data reports on postoperative recurrence monitoring of HCC. There was currently a lack of kits in the clinic that use fluorescence PCR technology to detect cfDNA methylation in blood for HCC diagnosis.

Through the analysis of Illumina HumanMethylation450 BeadChip microarray data from the TCGA and GEO databases, Liang et al. identified the potential diagnostic value of combining GNB4 and Riplet as DNA methylation markers for HCC detection [23]. In their study, the combination of circulating tumor cell counts and the determination of GNB4/Riplet methylation yielded an AUC value of 0.98, demonstrating a sensitivity of 88.2% and a specificity of 100% for HCC diagnosis [23]. In addition, Kim et al. reported that the combination of Riplet (also known as RNF135) and LDHB as DNA methylation markers displayed a sensitivity of 57% and a specificity of 94.2% for HCC diagnosis [24]. Furthermore, in our previous study, we screened seven DNA methylation markers based on the analysis of differentially methylated CpG sites from the TCGA and GEO databases. This was subsequently verified with the in‐house WGBS data from 12 pairs of HCCs and NATs. As a result, GNB4 and Riplet were ultimately identified as the optimal candidate markers for HCC diagnosis. The combination of GNB4/Riplet exhibited excellent discrimination between HCCs and controls in 64 tissue samples and 470 plasma samples [25]. As for plasma samples, it demonstrated a diagnosis sensitivity of 84.32%, specificity of 91.92%, and AUC value of 92.51% for all stages of HCC, as well as diagnosis sensitivities of 75.76% and 93.55% for stages I and II HCC, respectively [25]. This study aimed to evaluate the analytical and clinical diagnostic performance of the GNB4 and Riplet gene methylation kit for detecting HCC. At the same time, it will explore the performance of this kit for the postoperative monitoring of HCC.

## 2 Methods

### 2.1 Study Population

A total of 1055 participants from the First Affiliated Hospital of Zhengzhou University were included in the study between June 2022 and October 2022. Of these, 214 patients with primary HCC met the inclusion criteria: they were ≥ 18 years of age, had a clinical diagnosis of HCC and had not received surgery, radiotherapy, or chemotherapy; patients with other malignancies were excluded. A total of 25 HCC patients underwent surgical resection of HCC. Two hundred seventy six patients with chronic liver disease (CLD) who were considered at high risk for HCC received HCC surveillance but were not diagnosed with HCC. Additionally, 258 healthy individuals with no clinical symptoms of liver disease or history of cancer were enrolled. Two hundred eighty two patients with interfering cancers (pancreatic cancer, *n* = 15; gastric cancer, *n* = 34; esophagus cancer, *n* = 22; breast cancer, *n* = 53; colorectal cancer, *n* = 43; lung cancer, *n* = 52; gallbladder carcinoma and cholangiocarcinoma, *n* = 23; bladder cancer, *n* = 40). This clinical trial was approved by the Ethics Committee of the First Affiliated Hospital of Zhengzhou University (2022‐KY‐0631‐002) and registered at ClinicalTrials.gov. The clinical characteristics of all participants were shown in Tables S1 and S2. The average age of patients with HCC, patients with CLD, and healthy individuals was 59, 51, and 39 years old, respectively. The percentages of male HCC patients, male CLD patients, and male healthy individuals were 78.0%, 68.5%, and 59.7%, respectively. In addition, the majority of the HCC and CLD patients were found to be infected with hepatitis viruses (HCC: 71.0%; CLD: 61.2%), to have cirrhosis (HCC: 77.1%; CLD: 83.0%), and to have Child‐Pugh A/B scores (HCC: 84.6%; CLD: 56.2%). All participants signed an informed consent form and all data were collected in accordance with the tenets of the Declaration of Helsinki. A total of 10 mL blood was collected from all participants through the venipuncture upon enrollment into the group. The HCC patients were classified into early (stage I–II) and late (stage III–IV) stages based on the China Liver Cancer Staging System (CNLC) [6].

### 2.2 Cut‐Off Value

The 214 cases of HCC and 534 non‐HCC samples were randomly divided into training and validation sets at a 3:2 ratio. The clinical characteristics of the study participants were presented in Table S1. The training set contained 123 HCC and 326 non‐HCC samples, while the validation set contained 91 HCC and 208 non‐HCC samples. In the training set, the Ct cut‐off value of the reference gene β‐actin was calculated using the percentile method. Since 95% and 99% quantiles showed good repeatability in detection results, and the sample concentrations met detection requirements, both could be used as the upper boundary values for the Ct value of the reference gene. With the 99% quantile, fewer samples were discarded. Therefore, the Ct value corresponding to the 99% percentile was chosen as the cut‐off value for β‐actin (Table S3). A cut‐off of 35 was selected for β‐actin, meaning that samples with β‐actin Ct values ≤ 35.0 were considered valid, while those with Ct values of β‐actin > 35.0 were considered invalid.

Three analytical methods were compared to determine the cut‐off values for GNB4 and Riplet in the DT‐HBT. The first and second methods both used logistic regression algorithms to construct diagnostic models for HCC using the GNB4 and Riplet genes, and the diagnostic performance of the model was evaluated using ROC analysis. The first method built the model using 2^−ΔΔCt^ values of GNB4 and Riplet, while the second used the Ct values. The third method used the 1/2 algorithm to classify samples based on Ct values of the target genes, that is, a sample was positive if the Ct value of a single gene was positive. The ROC analysis was performed on the training set samples to determine the cut‐off values for the target genes. This yielded cut‐off values of 43.3 and 43.7 for GNB4 and Riplet, respectively (Figure S1). A final cut‐off of 43 was chosen for both genes to be applied in 1/2 algorithm. The sensitivity, specificity and accuracy of the three analytical methods for HCC diagnosis were compared in the training set, and the method with the best performance was selected for validation in the validation set. The performance was calculated using the following formulas:

$$\text{Sensitivity}=\frac{\text{True positive}}{\text{True positive}\;+\;\text{False negative}}\times 100$$

$$\text{Specificity}=\frac{\text{True negative}}{\text{True negative}\;+\;\text{False positive}}\times 100$$

$$\text{Accuracy}=\frac{\text{True positive}+\text{True negative}}{\text{True positive}\;+\;\text{False negative}\;+\;\text{True negative}\;+\;\text{False positive}}\times 100$$

### 2.3 Limit of Detection

Simulated plasma samples of different concentrations were prepared by adding methylated DNA extracted from GNB4 and Riplet methylation positive cell line (HepG2) into different concentrations of GNB4 and Riplet methylation negative plasma matrices prepared using healthy human plasma. The methylation status of GNB4 and Riplet in each simulated sample was determined by Sanger sequencing, and the methylation ratio was determined by ddPCR. The concentrations of the simulated samples were: 1%, 10%, and 50% of GNB4 and Riplet methylation ratios at a nucleic acid concentration of 10 copies/μL, respectively; 0.1%, 1%, and 5% GNB4 and Riplet methylation ratios at a nucleic acid concentration of 100 copies/μL, respectively; and 0.01%, 0.1%, and 1% GNB4 and Riplet methylation ratios at a nucleic acid concentration of 1000 copies/μL, respectively. The methylation of GNB4 and Riplet in each sample was detected repeatedly 20 times using 3 batches of kit to determine the detection limit. Subsequently, plasma samples from HCC patients with GNB4 and Riplet methylation were used to verify the minimum detection limit of the kit determined in the initial study.

### 2.4 Precision Analysis

The precision of HCC gene methylation blood detection kit was evaluated following the Clinical and Laboratory Standards Institute (CLSI) document EP15‐A3 using five mixed plasma samples (including 1 negative sample, 1 strongly positive sample for GNB4 and Riplet methylation, 1 moderately positive sample for GNB4 and Riplet methylation, 1 weakly positive sample for GNB4 methylation, and 1 weakly positive sample for Riplet methylation) with known methylation status of GNB4 and Riplet genes through Sanger sequencing and determined methylation ratio through ddPCR. Both GNB4 and Riplet were unmethylated in the negative plasma sample. The strong positive sample indicated that the methylation ratio for both GNB4 and Riplet were greater than or equal to 20%. The moderately positive sample indicated that the methylation ratio for each target gene was between 5% and 20%. The weakly positive sample has a methylation ratio of less than 5% for the target gene. The samples came from the remaining clinical plasma samples.

Intra‐assay (repeatability) and inter‐assay precision: Three batches of kits were used to detect the methylation of target and internal reference genes in 5 mixed plasma samples using the ABI 7500 fluorescence quantitative PCR instrument. Each sample was tested 10 times repeatedly using three batches of kits by the same operator under the same environmental conditions and time period. Intra‐assay precision was assessed by the coefficient of variations (CV) values of 10 repeated test results for each sample in each kit. Inter‐assay precision was assessed by the CV values of the 30 (10 times × 3 batches) test results for each sample obtained by the operator using three batches of kits. CV = Standard Deviation / Mean × 100%, the CV value ≤ 5% indicated a small degree of variance, and meeting criterion for both intra‐assay and inter‐assay precision.

Inter‐day precision and inter‐laboratory precision: Two operators (A and B) were arranged to use different instruments (same ABI 7500 model) in different laboratories, and use the same batch of kit to detect the methylation of target and internal reference genes in the samples once per day for 20 consecutive days. Since both parties conducted testing in different laboratories using different instruments, the precision between personnel is the same as that between equipment and laboratories. The inter‐day precision was assessed by the CV value of 20 test results for each sample using the same batch of kit by a single person over 20 consecutive days. The inter‐laboratory precision was assessed by the CV value of 40 test results (20 times × 2 persons) using the same batch of kit by two individuals on each sample for 20 days.

### 2.5 Sanger Sequencing

The cell‐free DNA (cfDNA) was extracted from plasma samples and then bisulfite‐converted for further use. The methylation status of GNB4 and Riplet in plasma samples was evaluated by Sanger sequencing. Briefly, the PCR assay was conducted with a volume of 40 μL containing 20 μL of cfDNA template and other essential components such as 10 × HA PCR buffer (4 μL), 2.5 mM dNTPs (3.2 μL), primers (2 μL, Sangon Biotech, China), and taq DNA polymerase (0.5 μL, TianGen Biotech, China). The primers for GNB4 and Riplet were as follows: forward primer (GNB4), 5′‐CGGGAAGTGTTTGCGTCG‐3′; reverse primer (GNB4), 5′‐AACTCCCTCCAAATAAAGGACG‐3′; forward primer (Riplet), 5′–GCGAAGTGAGTGAGTGAGTGAGTT‐3′, reverse primer (Riplet), 5′‐GAATATTTTATAAAACCTCCCCCAA‐3′. PCR amplification was performed on an ABI7500 Real Time PCR instrument (ThermoFisher, USA), comprising a pre‐denaturation at 95°C for 5 min, followed by 35 cycles of denaturation at 95°C for 30 s, annealing at 55°C for 30 s, and elongation at 72°C for 30 s, with a final elongation step at 72°C for 10 min. PCR products were purified and sequenced using the ABI 3131 platform (Sangon Biotech, China). The amplicons in the DT‐HBT are both within the amplicons for Sanger sequencing. As previously described, GNB4 and Riplet‐specific methylation primers and MGB probes detected eight and six key CpG sites in DT‐HBT amplicons, respectively [25]. According to the results of Sanger sequencing, a sample is considered methylation positive if more than half of the key CpG sites in the sample are methylated (i.e., the Sanger sequencing result of a CpG site is CG or CG and TG). A sample is considered methylation negative if less than or equal to half of the key CpG sites are unmethylated (i.e., the Sanger sequencing result of a CpG site is TG).

### 2.6 ddPCR (Digital Droplet PCR)

A ddPCR‐based approach was used to determine the concentration and the methylation ratio of the target genes. The ddPCR D3200 platform (Pilot Gene Technology, Hangzhou, China), consisting of a droplet generator, a PCR amplifier and a biochip reader, was used for droplet generation, PCR amplification and data analysis according to the manufacturer's instructions. A total volume of 16 μL of PCR mix was prepared, including primers (0.32 μL for each gene) and probes (0.048 μL for each gene) specific for GNB4, Riplet and ACTB, respectively, 2 μL of bisulfite‐converted cfDNA template, 1.6 μL of the 10 × HA PCR buffer, 1.3 μL of 2.5 mM dNTPs and 0.32 μL of High Affinity HotStart Taq DNA Polymerase (TianGen, China). The sequence of primers and probes was the same as for the DT‐HBT and was provided previously [25]. Single droplets were produced by loading the PCR mix onto the chip and running it on a droplet generator. PCR was then performed under the following conditions 95°C for 5 min, followed by 45 cycles at 95°C for 15 s and 60°C for 30 s. After PCR amplification, the chip was scanned and analyzed using a biochip reader. The results of the ddPCR detection include the copy numbers of ACTB, methylated GNB4 and methylated Riplet. Since ACTB, GNB4 and Riplet all exist as single copies in the human genome, the copy number of ACTB represents the concentration of the genome (i.e., the sum of the copy numbers of methylated and unmethylated GNB4). The ratio of a methylated gene was calculated as the percentage of the copy number of the methylated gene in the copy number of the genome.

### 2.7 ROC Analysis

The R package “pROC” was used to perform ROC curve analysis and estimate the AUC and 95% confidence interval (CI) of the model. The maximum Youden index, calculated using the following formula, was used to determine the optimal sensitivity and specificity:

Youden index=sensitivity+specificity−1.

### 2.8 Extraction of DNA From Plasma

Plasma was obtained from whole blood samples by centrifugation, followed by extraction of cfDNA from the plasma using the Plasma cfDNA Extraction Kit (AA16, Wuhan Ammunition, Wuhan, China). Briefly, to the 2 mL extracted plasma, 100 μL proteinase K, 2 mL lysis‐binding solution LBB, and 40 μL magnetic beads were added. The mixture was thoroughly mixed and incubated at room temperature for 30 min. At the end of the incubation period, magnetic beads with adsorbed cfDNA were collected. The beads were then washed twice with WB1 wash solution and WB2 rinse solution. Finally, the cfDNA was eluted with 80–150 μL of eluent TE.

The Ct value of β‐actin was used to assess the quality of the cfDNA. Valid cfDNA samples were those with a Ct value of β‐actin below 35. The extracted cfDNA required immediate bisulfite conversion.

### 2.9 Bisulfite Conversion and Purification

The cfDNA was treated with sodium bisulfite to selectively convert unmethylated cytosines into uracils, while leaving methylated cytosines unaffected. The Plasma cfDNA Conversion Kit (AA20, Wuhan Ammunition, Wuhan, China) was used to convert cfDNA. Briefly, to prepare the sample, add 5 μL of protection solution and 110 μL of transformation solution to 35 μL of cfDNA solution. Shake and mix the solution for 5 s. After centrifugation, immediately place the sample in a PCR machine and amplify it using the following conditions: 95°C for 10 min, 64°C for 60 min, and 4°C for 3 min to 12 h. The transformation products were incubated with a binding solution and magnetic bead suspension at room temperature for 15 min. After incubation, the magnetic beads were washed with washing buffer. The bisulfite‐converted cfDNA adsorbed by the magnetic beads was eluted by adding eluent TE after washing. The real‐time fluorescence quantitative PCR was performed immediately on the eluted bisulfite‐converted DNA.

### 2.10 Cell Lines

HepG2 cells were obtained from the China Center for Type Culture Collection (CCTCC). The cells were cultured in DMEM (11 960 044, Thermo Fisher, MA, USA) supplemented with 10% fetal bovine serum (12 484 010, Thermo Fisher, MA, USA). HepG2 cells were used as a positive control for the differential methylation region of the GNB4 and Riplet genes.

### 2.11 Methylation‐Specific PCR and the Data Analysis

PCR amplification of bisulfite‐converted cfDNA was performed in a 50 μL system. The reaction mixture contained 40 μL of template DNA, 6 μL of PCR reaction I (PCR buffer and dNTP), 3.4 μL of PCR reaction II (primers and probes), and 0.6 μL of Taq enzyme. The reaction conditions were as follows: 95°C for 5 min, 95°C for 15 s, and 60°C for 30 s, 45 cycles.

After completing PCR, a baseline was established, and the Ct values of both the target and reference genes were recorded. Methylation levels (2^−ΔΔCt^) for each gene were then calculated in every sample. ΔΔCt = (Ct_target gene_ − Ct_internal reference_) _sample_ − (Ct_target gene_ − Ct_internal reference_) _positive control_.

### 2.12 Statistical Analysis

All statistical analyses were performed using GraphPad Prism 8.0. Two groups of data were compared using the Wilcoxon rank‐sum test, multiple groups of data were compared using the Kruskal–Wallis rank‐sum test, and categorical variables were compared using the chi‐square test. Differences with a *p* < 0.05 were considered statistically significant.

## 3 Results

**FIGURE 1:**
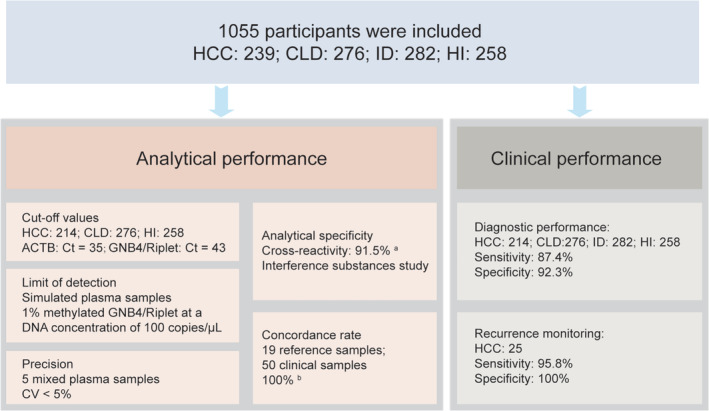
Study overview. The overall strategy for the assessment of analytical and clinical performance of the DT‐HBT is illustrated. Hepatocellular carcinoma, chronic liver disease, interfering disease (malignancies other than hepatocellular carcinoma), and healthy individuals are abbreviated as HCC, CLD, ID, and HI, respectively. “a” indicated the specificity of the DT‐HBT in identifying ID samples, which eight other types of cancer. “b” indicated the concordance rate between the results of the DT‐HBT and the reference samples, as well as the clinical samples.

The overview of this study was demonstrated in Figure 1. A total of 1055 participants were included in this study to evaluate the analytical and clinical performance of the DT‐HBT for HCC detection.

### 3.1 Determine the cut‐Off Value and Algorithm of the DT‐HBT

The cut‐off value of the DT‐HBT was studied in plasma cohorts. In the training set, the three methods were compared for calculating the diagnostic performance of DT‐HBT for HCC (Table 1). The results showed that Logistic (Ct) had the same sensitivity as Logistic (2^−ΔΔCt^), but higher specificity, AUC, and accuracy than Logistic (2^−ΔΔCt^). The 1/2 algorithm method, which had the same specificity as Logistic (Ct) but slightly lower sensitivity, had the second highest detection accuracy after Logistic (Ct).

**TABLE 1:** The performance of the three methods in the training set.

Target	GNB4 and Riplet		
Indicator	Logistic (2^−ΔΔCt^)	Logistic (Ct)	Ct
Specificity	89.9%	92.3%	92.3% (301/326)
Sensitivity	88.6%	88.6%	86.2% (106/123)
AUC (95% CI)	91.4% (88.4–95.3)	92.7% (90.1–95.2)	/
Accuracy	89.5% (402/449)	91.3% (410/449)	90.6% (407/449)
Comparisons	HCC vs. non‐HCC		

*Note:* HCC, *n* = 123; non‐HCC, *n* = 326.

In the validation set, the Ct values of β‐actin were less than 35 for all 299 samples, indicating that these were all valid samples. The cut‐off values obtained from the training set using the three methods were further used to judge the samples and calculate the sensitivity of the kit for detecting HCC, which was 87.9% for all methods. The specificity in non‐HCC samples and overall accuracy calculated using the Logistic (2^−ΔΔCt^) method were the lowest, at 86.1% and 86.6%, respectively. The Logistic (Ct) and 1/2 algorithm methods had the same performance, with specificity and accuracy of 93.9% and 91.6%, respectively (Table 2). Considering the clinical applicability, the 1/2 algorithm method was used for result judgment. Therefore, the 1/2 algorithm method was chosen for the DT‐HBT, that is, when the detection result of GNB4 or Riplet was Ct ≤ 43, the sample result was positive; when the detection result of GNB4 or Riplet was Ct > 43, the sample result was negative.

**TABLE 2:** The performance of the three methods in the validation set.

Target	GNB4 and Riplet		
Indicator	Logistic (2^−ΔΔCt^)	Logistic (Ct)	Ct
Specificity	86.1% (179/208)	93.3% (194/208)	93.3% (194/208)
Sensitivity	87.9% (80/91)	87.9% (80/91)	87.9% (80/91)
Accuracy	86.6% (259/299)	91.6% (274/299)	91.6% (274/299)
Cut‐off	0.135	0.129	43
Comparisons	HCC vs. non‐HCC		

*Note:* HCC, *n* = 91; non‐HCC, *n* = 208.

### 3.2 Determine the LOD of the DT‐HBT

The LOD was served as an important indicator for the detection ability of the kit. As shown in Table 3, at a DNA concentration of 10 copies/μL, the kit achieved a detection rate of 100% for samples with methylation ratios of 10% and 50%, whereas samples with a 1% ratio were mostly < 95%. Detection rates for 1% and 5% ratios were 100% at 100 copies/μL, while 0.1% ratios were mostly < 100%. For a concentration of 1000 copies/μL, detection rates were 100% for 0.1% and 1% ratios, while 0.01% ratios were mostly < 100%. Considering that most plasma samples have a nucleic acid concentration of under 100 copies/μL, this kit demonstrates a LOD of 1% methylated DNA for both GNB4 and Riplet at a DNA concentration of 100 copies/μL.

**TABLE 3:** LOD of the DT‐HBT using different concentrations of reference samples.

Reference	Reagent batch	Nucleic acid concentration								
		10 copies/μL			100 copies/μL			1000 copies/μL		
		1%	10%	50%	0.10%	1%	5%	0.01%	0.10%	1%
Reference 1 (GNB4^+^ Riplet^+^)	Batch 1	95% (19/20)	100% (20/20)	100% (20/20)	100% (20/20)	100% (20/20)	100% (20/20)	100% (20/20)	100% (20/20)	100% (20/20)
	Batch 2	90% (18/20)	100% (20/20)	100% (20/20)	85% (17/20)	100% (20/20)	100% (20/20)	95% (19/20)	100% (20/20)	100% (20/20)
	Batch 3	95% (19/20)	100% (20/20)	100% (20/20)	95% (19/20)	100% (20/20)	100% (20/20)	100% (20/20)	100% (20/20)	100% (20/20)
Reference 2 (GNB4^+^ Riplet^−^)	Batch 1	65% (13/20)	100% (20/20)	100% (20/20)	85% (17/20)	100% (20/20)	100% (20/20)	70% (14/20)	100% (20/20)	100% (20/20)
	Batch 2	85% (17/20)	100% (20/20)	100% (20/20)	70% (14/20)	100% (20/20)	100% (20/20)	85% (17/20)	100% (20/20)	100% (20/20)
	Batch 3	60% (12/20)	100% (20/20)	100% (20/20)	85% (17/20)	100% (20/20)	100% (20/20)	85% (17/20)	100% (20/20)	100% (20/20)
Reference 3 (GNB4^−^ Riplet^+^)	Batch 1	65% (13/20)	100% (20/20)	100% (20/20)	75% (15/20)	100% (20/20)	100% (20/20)	80% (16/20)	100% (20/20)	100% (20/20)
	Batch 2	85% (17/20)	100% (20/20)	100% (20/20)	75% (15/20)	100% (20/20)	100% (20/20)	75% (15/20)	85% (17/20)	100% (20/20)
	Batch 3	80% (16/20)	100% (20/20)	100% (20/20)	80% (16/20)	100% (20/20)	100% (20/20)	75% (15/20)	100% (20/20)	100% (20/20)

*Note:* GNB4^+^ Riplet^−^ indicated samples positive for GNB4 and Riplet methylation; GNB4^+^ Riplet^−^ indicated that the sample is positive for GNB4 methylation and negative for Riplet methylation; GNB4^−^ Riplet^+^ indicated that the sample is negative for GNB4 methylation and positive for Riplet methylation.

### 3.3 Assessing the Precision of the DT‐HBT

**FIGURE 2:**
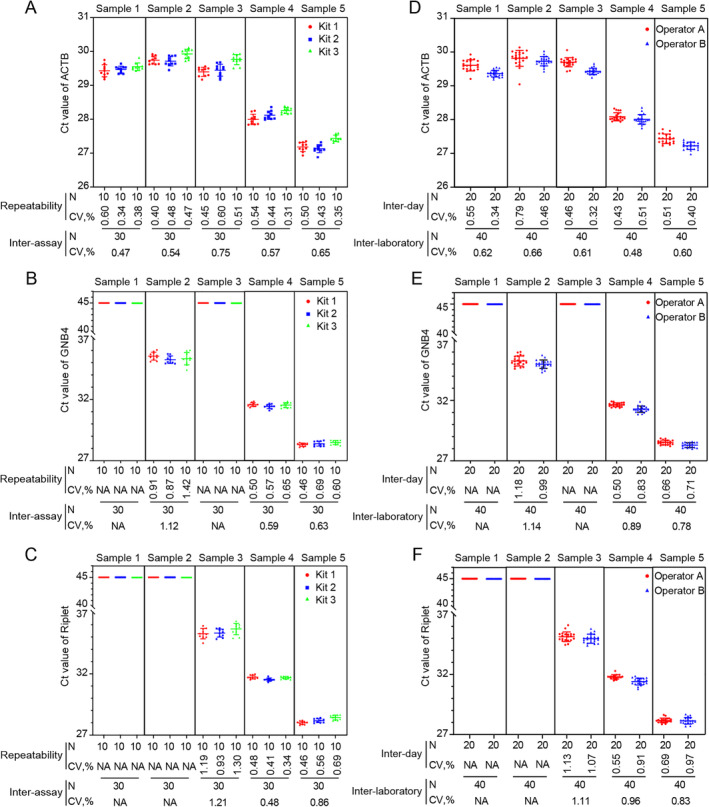
The DT‐HBT exhibit excellent precision. Repeatability and inter‐assay precision of the kit were assessed for β‐Actin (A), GNB4 (B), and Riplet (C); Inter‐day and inter‐laboratory precision were assessed for β‐actin (D), GNB4 (E), and Riplet (F). N: the number of tests; CV: the coefficient of variability; five samples were tested: Sample 1 (GNB4^−^ Riplet^−^), Sample 2 (GNB4^+^ Riplet^−^), Sample 3 (GNB4^−^ Riplet^+^), Sample 4 (GNB4^++^ Riplet^++^), and Sample 5 (GNB4^+++^ Riplet^+++^); The methylation status is indicated as: “−” = negative (unmethylated), “+” = weakly positive (methylated), “++” = moderate positive, “+++” = strongly positive.

To assess the reproducibility of kit for HCC detection, the precision was calculated. The CV for repeatability testing was below 1.5% for each batch of the kit after 10 duplicate tests of five plasma samples with different methylation statuses. The CV values of 30 tests on each sample using three batches of the kits were also less than 1.5%. This indicated satisfactory repeatability and inter‐assay precision of the kit (Figure 2A–C). To evaluate the inter‐day and inter‐laboratory precision, two different operators conducted testing in different laboratories using different instruments on separate days. The CV values were calculated for each sample tested over 20 consecutive days by both operators using the same batch of kit. The CV values for each operator were less than 1.5% over 20 days and CV values across 40 total tests by operators A and B were all less than 1% per sample. This indicated that the inter‐day and inter‐laboratory precision of the kit meet the requirements (Figure 2D–F).

### 3.4 Analytical Specificity of the DT‐HBT Was Evaluated

**FIGURE 3:**
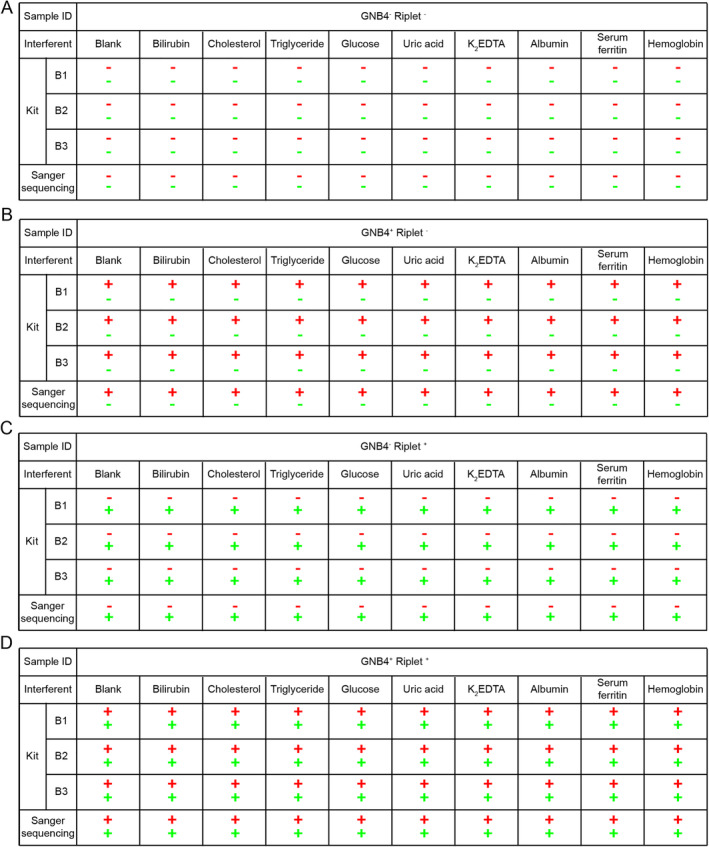
Verification of the anti‐interference ability of the DT‐HBT against interfering substances contained in the sample. The clinical samples with different methylation patterns were detected by different batches of kits and Sanger sequencing. (A) The clinical sample was negative for methylation of both genes, GNB4^−^ Riplet^−^; (B) the clinical sample was positive for methylation of GNB4 only, GNB4^+^ Riplet^−^; (C) the clinical sample was positive for methylation of Riplet only, GNB4^−^ Riplet^+^; (D) the clinical sample was positive for methylation of both GNB4 and Riplet, GNB4^+^ Riplet^+^. The following interfering substances were added to each sample: 0.4 mg/mL bilirubin, 12 mg/mL cholesterol, 20 mg/mL triglycerides, 10 mg/mL glucose, 0.35 mg/mL uric acid, 20 mg/mL K2EDTA, 50 mg/mL albumin, 2 mg/mL serum ferritin, and 17.5 mg/mL hemoglobin. B1–B3 indicated the three batches of kits used; red represented the result of GNB4, green represented the result of Riplet, “−” indicated negative methylation, “+” indicated positive methylation.

The analytical specificity study includes cross‐reactivity and interference studies. In the cross‐reactivity study, this study recruited 282 patients with other types of cancer to use as interfering diseases to test the specificity of the kit for HCC detection. The results showed that the specificity was more than 80% for all interfering cancers, except for gallbladder carcinoma and cholangiocarcinoma. Overall, the specificity for all interfering cancers was 91.5% (Table 4). In the interference study, four clinical samples with different methylation patterns were collected (GNB4^−^ Riplet^−^, GNB4^+^ Riplet^−^, GNB4^−^ Riplet^+^, and GNB4^+^ Riplet^+^), and different interfering substances were added to the samples for testing. The test results of the kit on the sample were consistent with the Sanger sequencing results, indicating that the methylation status of the target gene was not affected (Figure 3A–D). Therefore, the presence of these substances (0.4 mg/mL bilirubin, 12 mg/mL cholesterol, 20 mg/mL triglyceride, 10 mg/mL glucose, 0.35 mg/mL uric acid, 20 mg/mL K2EDTA, 50 mg/mL albumin, 2 mg/mL transferrin, 17.5 mg/mL hemoglobin) in the samples did not affect the test results of the kit.

**TABLE 4:** The specificity of the DT‐HBT for interfering diseases.

Interfering diseases	Specificity
Pancreatic cancer	80.0% (12/15)
Gastric cancer	85.3% (29/34)
Esophagus cancer	90.0% (20/22)
Breast cancer	100% (53/53)
Colorectal cancer	86.0% (37/43)
Lung cancer	100% (52/52)
Gallbladder carcinoma and cholangiocarcinoma	65.2% (15/23)
Bladder cancer	100% (40/40)
Total	91.5% (258/282)

### 3.5 Coincidence Rate of the DT‐HBT Was Evaluated

**FIGURE 4:**
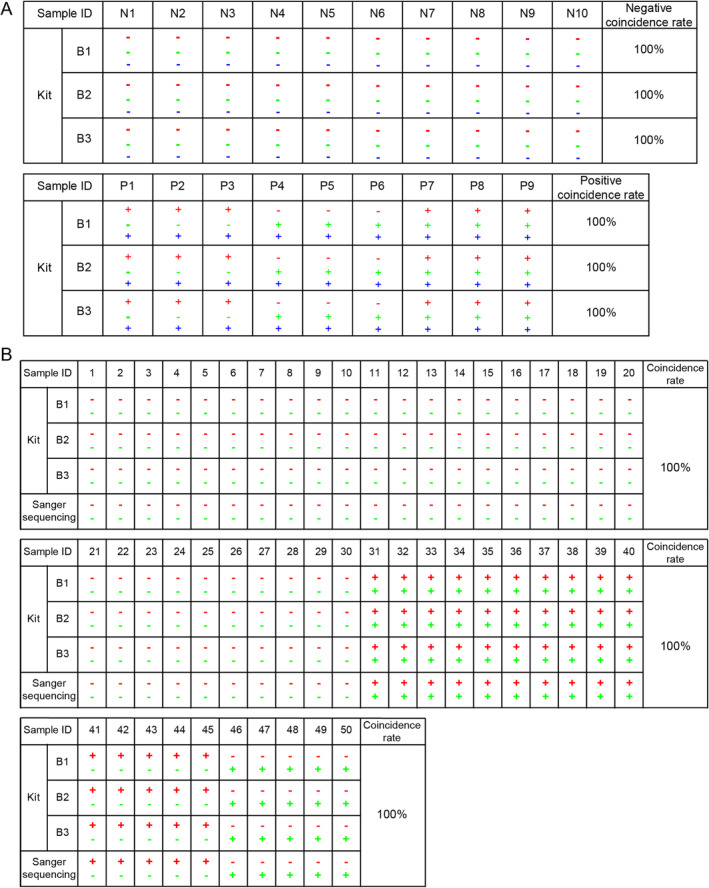
The coincidence rate of the DT‐HBT in detecting reference and clinical samples. (A) The coincidence rates of three batches of kits for detecting negative and positive reference samples were calculated. N1–N10 were 10 negative reference samples, P1–P9 were 9 positive reference samples, B1–B3 were the three batches of kits; Red represented the result of GNB4, green represented the result of Riplet, blue represented kit results with “−” indicating methylation negative and “+” indicating methylation positive. (B) The consistency between the results from the three batches of kits and Sanger sequencing was compared in 50 clinical samples. Samples 1–30 were 30 non‐HCC samples, and 31–50 were 20 HCC samples.

The coincidence rate of the kit was assessed by calculating the positive and negative coincidence rates between the kit results and expected results for reference and clinical samples. The kit was tested on 10 negative reference samples (N1–N10) and 9 positive reference samples (P1–P9). The negative and positive coincidence rates were both 100% for the reference samples (Figure 4A). The rates were further tested on 50 clinical plasma samples (HCC, *n* = 20; non‐HCC, *n* = 30). The positive and negative coincidence rates between the kit results and Sanger sequencing results were both 100% for the clinical samples (Figure 4B).

### 3.6 Performance of the DT‐HBT for HCC Diagnosis and Postoperative Recurrence Monitoring

Further analysis of the diagnostic performance of the dual‐target HCC detection kit on primary HCC revealed that the sensitivity of this kit was 81.7% for primary HCC stages I, 90.0% for primary HCC stages II, 92.0% for primary HCC stages III‐VI, and 87.4% for all stages primary HCC. The specificity was 86.6% in the CLD population and 99.2% in the healthy population (Table 5). As shown in Table S4, age, sex, CNLC stage, hepatitis virus infection, cirrhosis, and Child‐Pugh score of HCC patients did not affect the diagnostic sensitivity of the DT‐HBT. Moreover, the age, sex, hepatitis virus infection, cirrhosis, and Child‐Pugh score of CLD patients, as well as the age and sex of healthy individuals, did not have an impact on the diagnostic specificity of the DT‐HBT (Tables S5 and S6).

**TABLE 5:** Performance of the DT‐HBT for primary HCC diagnosis.

Target	Sensitivity	Specificity	Comparisons
GNB4 + Riplet	81.7% (67/82)	/	Stage I
	90.0% (36/40)	/	Stage II
	94.6% (80/87)	/	Stage III
	76.9% (10/13)	/	Stage IV
	87.4% (187/214)	/	All stages
	/	86.6% (239/276)	CLD
	/	99.2% (256/258)	Normal
	/	91.5% (258/282)	Interfering diseases (ID)
	/	92.3% (752/816)	CLD and normal and ID

*Note:* The number of samples was the sum of the training set and validation set. All stages primary HCC, *n* = 214; stage I, *n* = 82; stage II, *n* = 40; stage III‐IV, *n* = 87; unknown stage HCC, *n* = 5; non‐HCC, *n* = 534; CLD, *n* = 276; normal, *n* = 258.

Hepatectomy is currently recommended as a radical therapy for HCC in clinical guidelines. Postoperative recurrence monitoring can prolong the survival of patients undergoing hepatectomy. By analyzing the plasma test results of 25 hepatectomy patients, the sensitivity, specificity, and accuracy of the dual‐target HCC detection kit for detecting postoperative recurrence were found to be 95.8% (23/24), 100.0% (1/1), and 96.0% (24/25), respectively (Table 6). This indicated that Riplet and GNB4 methylation have potential for postoperative recurrence monitoring in hepatectomy patients.

**TABLE 6:** The performance of the DT‐HBT for monitoring postoperative recurrence of HCC patients after hepatectomy.

GNB4 + Riplet	Real recurrence	Real no. recurrence	
Predict recurrence	23	0	
Predict no recurrence	1	1	Totals
Totals	24	1	25
Correct	23	1	24
Sensitivity	95.8%		
Specificity		100%	
Accuracy			96.0%

## 4 Discussion

HCC, which is a disease with high incidence and mortality rates, has occurrence and progression that are processes of accumulative epigenetic alterations [26]. The application of cfDNA methylation markers in HCC diagnosis and postoperative monitoring had greatly improved the survival of HCC patients. Based on previous studies, we implemented the application of GNB4 and Riplet gene methylation in HCC diagnosis, developed a blood test for HCC, and comprehensively evaluated its analytical and clinical performance.

Currently, the performance evaluation of in vitro diagnostic reagents on an international level is usually based on relevant standards of the CLSI in the United States, which are also recommended evaluation standards by the U.S. FDA [27]. The analytical performance evaluation of the in vitro HCC diagnostic kit in this study comprised the cut‐off value, LOD, precision, analytical specificity, and coincidence rate.

The LOD is a crucial indicator for assessing in vitro diagnostic kits as it denotes the analytical sensitivity of the kit [28]. The DT‐HBT showed an exceptionally low LOD, measuring only 1% of the target gene methylation ratio at a nucleic acid concentration of 100 copies/μL. The methylation ratio of cfDNA in the plasma of patients with BCLC A was 15.25% [22], which was higher than the LOD of this kit, indicating that DT‐HBT has a high analytical sensitivity to detect hypomethylation signals in early cancers. Therefore, it is suitable for early detection of HCC.

The precision was another important indicator reflecting the stability and accuracy of kit detection. It referred to the degree of consistency between results obtained by replicate measurements on the same or similar objects under specified conditions. The precision included repeatability, intermediate precision, and reproducibility [29]. Repeatability is often referred to as precision under repeatable conditions, which represents measurement conditions that remain essentially unchanged. Reproducibility, also known as inter‐laboratory precision, indicates maximum variation in measurement conditions. Intermediate precision lies between repeatability and reproducibility, covering the kit's inter‐assay and inter‐day precision. When detecting weak positive samples, the CV value of β‐actin should be lower than that of GNB4 and Riplet. Increasing the changes to the detection conditions resulted in higher CV values for GNB4 and Riplet. In contrast, the CV value of β‐actin had smaller variations. These results suggest that the concentration of methylation in the sample and detection conditions can influence the detection stability of the DT‐HBT. Furthermore, the target genes were more affected than the reference gene. Although the factors greatly influenced the DT‐HBT detection results, all CV values were below 1.5%, meeting the prespecified criteria for clinical application.

To select a proper algorithm, three analytical methods were conducted in the study. The findings demonstrated that the Ct value analysis methods achieved higher accuracy compared to 2^−ΔΔCt^ (91.3%/90.6% vs. 89.5%). Moreover, the clinical performance obtained from the two Ct value methods indicated that the logistic regression algorithm had greater sensitivity and accuracy than the 1/2 algorithm (sensitivity: 88.6% vs. 86.2%; accuracy: 91.3% vs. 90.6%). However, when considering clinical applicability and generalizability, interpreting the test data directly may decrease errors after data processing, and the disparity in performance between the two methods was minimal. As a result, the DT‐HBT has adopted the 1/2 algorithm to determine the test result: the test is considered positive if the target gene detection result is Ct ≤ 43, and negative if it is Ct > 43. These findings were additionally confirmed in the validation cohort, where both Ct values approaches exhibited superior performance to 2^−ΔΔCt^ method, with comparable clinical performance between the two Ct value methods.

Analytical specificity studies encompassed cross‐reactivity and interference test [30]. For cross‐reactivity studies, the kit demonstrated an overall analytical specificity of 91.5% for interference cancers. However, specificity dropped to 65.2% for gallbladder and cholangiocarcinoma. The liver was positioned anatomically adjacent to the gallbladder and bile ducts, and there was frequent mutual invasion and metastasis between HCC, gallbladder, and cholangiocarcinoma [31]. In clinical practice, regardless of whether a patient was suspected of liver cancer or gallbladder and cholangiocarcinoma, doctors would examine the liver, gallbladder, and bile ducts at the same time to determine whether there is cancer. Therefore, the detection of gallbladder and cholangiocarcinoma by the DT‐HBT would facilitate the early diagnosis and treatment of patients without increasing the diagnostic and therapeutic burdens of patients.

Based on previous studies, this study demonstrated that the DT‐HBT meets the analytical performance requirements and established the criteria for determining results. Building on this, the study further evaluated the clinical performance of the DT‐HBT using these criteria. The results showed a sensitivity of 81.7% for I‐stage HCC detection, 90.0% for II‐stage HCC, 92.0% for late‐stage HCC, and an all‐stage sensitivity of 87.4%. The specificity achieved 86.6% in CLD population and 99.2% in healthy population, outperforming the majority of comparable products available in the market [22, 32]. AFP is the most commonly used commercial blood test for the detection and surveillance of HCC. However, its sensitivity and specificity for HCC detection have been reported to be 60% and 84%, respectively, at a cut‐off of 20 ng/mL [33]. The low clinical performance might limit its utility. With a sensitivity of 84.4% for early HCCs and an overall specificity of 92.3% (Table 5), the DT‐HBT may be capable of detecting more HCC patients at a resectable stage. Recently, DNA methylation markers have been extensively studied for the detection of HCC. Xu et al. reported that a diagnostic prediction model containing 10 markers and using targeted bisulfite sequencing to interrogate their methylation levels achieved a sensitivity of 83.3% and a specificity of 90.5% in the validation dataset of 383 HCC and 275 normal samples [34]. Compared to the 10 markers model, the diagnostic performance of the DT‐HBT is not inferior, but it is much more cost‐effective and convenient to perform. The mt‐HBT, which incorporates three methylation markers (HOXA1, TSPYL5, and B3GALT6), AFP and sex, demonstrated an overall sensitivity of 88%, early stage sensitivity of 82% and specificity of 87% in an independent cohort consisting of 156 HCC cases and 245 controls [35]. In our study, the DT‐HBT exhibited a similar sensitivity to the mt‐HBT, but with higher specificity and without the need for a complex algorithm. In addition, based on the qMSP method, mSEPT9 has been reported to be able to discriminate HCC from controls with a sensitivity of 64%–88% and a specificity of 68%–95% for early HCC sensitivity of 64%–88% and a specificity of 68%–95% for early HCC [36, 37]. Regardless of its large fluctuation in sensitivity, the mSEPT9 test is well known to detect colorectal cancer, we infer that the cross‐reactivity of mSEPT9 might be a limitation of it for HCC detection. Therefore, we conclude that the DT‐HBT, using only two methylation markers, demonstrated excellent performance in the detection of HCC.

Monitoring recurrence after hepatectomy was also a clinical focus, therefore this study collected 25 patients who underwent hepatectomy, among whom 24 had postoperative recurrence and 1 did not. The sensitivity of the DT‐HBT in detecting postoperative recurrence was 95.8%, the specificity was 100%, and the accuracy reached 96%. Since there was only 1 case without postoperative recurrence, the results for the specificity of the kit need further validation.

However, the study had its limitations. First, it lacked multi‐center clinical trials to validate the clinical performance of the DT‐HBT. Second, the sample size was small in the recurrence monitoring after hepatectomy study. A larger sample size was needed to support the current findings.

In summary, based on previous studies, this study transformed the clinical application of GNB4 and Riplet gene methylation detection for HCC by developing the DT‐HBT for early HCC blood detection. It was found that the analytical performance of the DT‐HBT met the requirements. The sensitivity of the DT‐HBT reached 84.4% for early stage HCC and 95.8% for detecting recurrence in patients after hepatectomy. It can be used not only for early HCC detection, but also has potential for postoperative recurrence monitoring.

## Author Contributions

**Qiankun Yang:** conceptualization (equal), data curation (lead), formal analysis (lead), investigation (lead), methodology (lead), validation (lead), visualization (supporting), writing – original draft (equal). **Lanlan Dong:** conceptualization (equal), data curation (lead), formal analysis (lead), investigation (lead), methodology (lead), validation (lead), visualization (supporting), writing – original draft (equal). **Lianglu Zhang:** conceptualization (supporting), formal analysis (equal), investigation (equal), resources (equal), supervision (equal), writing – review and editing (supporting). **Wei Zhang:** data curation (equal), formal analysis (equal), investigation (equal), validation (supporting), writing – original draft (supporting). **Yan Zhang:** data curation (equal), formal analysis (supporting), validation (equal), writing – original draft (supporting). **Yue Huang:** data curation (supporting), formal analysis (supporting), visualization (equal), writing – original draft (supporting). **Huifang Jin:** data curation (supporting), investigation (supporting), project administration (equal), writing – review and editing (supporting). **Hao Yang:** data curation (supporting), investigation (supporting), project administration (supporting), validation (supporting), writing – original draft (supporting). **Xing Liu:** conceptualization (supporting), funding acquisition (equal), methodology (equal), project administration (equal), resources (equal), supervision (equal), writing – review and editing (equal). **Yanteng Zhao:** conceptualization (lead), funding acquisition (lead), methodology (equal), project administration (supporting), resources (equal), supervision (equal), writing – review and editing (equal).

## Ethics Statement

This study was approved by the Ethics Committee of the First Affiliated Hospital of Zhengzhou University (2022‐KY‐0631‐002). All human samples and clinical data were collected in accordance with the principles of the Helsinki Declaration.

## Consent

Each participant signed an informed consent form for the use of their tissues and blood samples.

## Conflicts of Interest

The authors declare no conflicts of interest.

## Supporting information


**Data S1.** Supporting information.

## Data Availability

Availability of data and materials. The datasets used and/or analyzed during the current study are available from the corresponding author upon reasonable request.
