# Supplementary material for: Analytical and Diagnostic Performance of a Dual‐Target Blood Detection Test for Hepatocellular Carcinoma
Source: Cancer Rep (Hoboken). 2024 Sep 26;7(9):e70017. doi: 10.1002/cnr2.70017 (PMC11425738; doi:10.1002/cnr2.70017)
Supplement: Supplementary file 1 — Data S1. Supporting information. [file CNR2-7-e70017-s001.docx]

**Supporting Information**

TABLE S1 The clinical characteristics of 214 HCC and 534 non-HCC individuals.

|  | HCC | | | CLD | | | Healthy | | |
| --- | --- | --- | --- | --- | --- | --- | --- | --- | --- |
|  | Training | Validation | All | Training | Validation | All | Training | Validation | All |
| Total, n | 123 | 91 | 214 | 168 | 108 | 276 | 158 | 100 | 258 |
| Age, years |  |  |  |  |  |  |  |  |  |
| Mean ± SD | 59 ± 12 | 60 ± 10 | 59 ± 11 | 52 ± 13 | 49 ± 14 | 51 ± 14 | 38 ± 12 | 39 ± 11 | 39 ± 12 |
| Gender |  |  |  |  |  |  |  |  |  |
| Male | 97 | 70 | 167 | 118 | 71 | 189 | 96 | 58 | 154 |
| Female | 26 | 21 | 47 | 50 | 37 | 87 | 62 | 42 | 104 |
| Hepatitis virus infection |  |  |  |  |  |  |  |  |  |
| Yes | 91 | 61 | 152 | 107 | 62 | 169 |  |  |  |
| No | 2 | 4 | 6 | 11 | 11 | 22 |  |  |  |
| Unknown | 30 | 26 | 56 | 50 | 35 | 85 |  |  |  |
| Cirrhosis |  |  |  |  |  |  |  |  |  |
| Yes | 96 | 69 | 165 | 133 | 96 | 229 |  |  |  |
| No | 3 | 7 | 10 | 5 | 2 | 7 |  |  |  |
| Unknown | 24 | 15 | 39 | 30 | 10 | 40 |  |  |  |
| Child-Pugh class |  |  |  |  |  |  |  |  |  |
| A/B | 103 | 78 | 181 | 93 | 62 | 155 |  |  |  |
| C | 5 | 6 | 11 | 23 | 22 | 45 |  |  |  |
| Unknown | 15 | 7 | 22 | 52 | 24 | 76 |  |  |  |
| CNLC stage |  |  |  |  |  |  |  |  |  |
| I-II | 63 | 48 | 111 |  |  |  |  |  |  |
| III-IV | 45 | 40 | 85 |  |  |  |  |  |  |
| Unknown | 15 | 3 | 18 |  |  |  |  |  |  |

TABLE S2 The clinical characteristics of 25 HCC patients undergoing hepatectomy.

| Characteristic | HCC |
| --- | --- |
| Total, n | 25 |
| Age, years |  |
| Mean ± SD | 57 ± 8 |
| Gender |  |
| Male | 20 |
| Female | 5 |
| Hepatitis virus infection |  |
| Yes | 22 |
| No | 3 |
| Cirrhosis |  |
| Yes | 5 |
| No | 20 |
| Child-Pugh class |  |
| A/B | 25 |
| C | 0 |
| CNLC stage |  |
| I-II | 24 |
| III-IV | 1 |

TABLE S3 Percentile analysis for Ct values of the reference gene β-actin in training set.

| Indicator | Percentile | |
| --- | --- | --- |
|  | 95% | 99% |
| Cut-off value | 32.43 | 34.59 |
| Excluded sample | 21 | 4 |

TABLE S4 Sensitivities of the DT-HBT for all HCC patients grouped by sex, age, CNLC stages, hepatitis virus infection status, cirrhosis status and Child-Pugh class.

|  | | N | Positive results (N) | Sensitivity (%) | Chi-square test/Fisher’s exact test, P value |
| --- | --- | --- | --- | --- | --- |
| Total | | 214 | 187 | 87.4 |  |
| Age | |  |  |  |  |
|  | ≤ 49 | 33 | 26 | 78.8 | P = 0.124 |
|  | 50-59 | 78 | 67 | 85.9 |  |
|  | 60-69 | 61 | 58 | 95.1 |  |
|  | ≥ 70 | 42 | 36 | 85.7 |  |
| Sex | |  |  |  |  |
|  | Male | 167 | 146 | 87.4 | P = 0.972 |
|  | Female | 47 | 41 | 87.2 |  |
| CNLC stage |  |  |  |  |  |
|  | Ⅰ | 82 | 67 | 81.7 | P = 0.055 |
|  | Ⅱ | 40 | 36 | 90 |  |
|  | Ⅲ | 74 | 70 | 94.6 |  |
|  | Ⅳ | 13 | 10 | 76.9 |  |
|  | Unknown | 5 | 4 | 80.0 |  |
| Hepatitis virus infection | |  |  |  |  |
|  | Yes | 152 | 129 | 84.9 | P = 0.162 |
|  | No | 6 | 5 | 83.3 |  |
|  | Unknown | 56 | 53 | 94.6 |  |
| Cirrhosis | |  |  |  |  |
|  | Yes | 165 | 144 | 87.27 | P = 0.625 |
|  | No | 10 | 8 | 80 |  |
|  | Unknown | 39 | 35 | 89.74 |  |
| Child-Pugh class | |  |  |  |  |
|  | A/B | 181 | 158 | 87.29 | P = 0.162 |
|  | C | 11 | 8 | 72.73 |  |
|  | Unknown | 22 | 21 | 95.45 |  |

TABLE S5 Specificities of the DT-HBT for participants with CLD.

|  | | N | Negative results (N) | Specificity (%) | Chi-square test, P value |
| --- | --- | --- | --- | --- | --- |
| Total | | 276 | 239 | 86.6 |  |
| Age | |  |  |  |  |
|  | ≤ 49 | 125 | 111 | 88.8 | P = 0.585 |
|  | 50-59 | 85 | 70 | 82.4 |  |
|  | 60-69 | 50 | 44 | 88.0 |  |
|  | ≥ 70 | 16 | 14 | 87.5 |  |
| Sex | |  |  |  |  |
|  | Male | 189 | 161 | 85.2 | P = 0.311 |
|  | Female | 87 | 78 | 89.7 |  |
| Hepatitis virus infection | |  |  |  |  |
|  | Yes | 169 | 144 | 85.2 | P = 0.410 |
|  | No | 22 | 21 | 95.5 |  |
|  | Unknown | 85 | 74 | 87.1 |  |
| Cirrhosis | |  |  |  |  |
|  | Yes | 229 | 197 | 86.0 | P = 0.265 |
|  | No | 7 | 5 | 71.4 |  |
|  | Unknown | 40 | 37 | 92.5 |  |
| Child-Pugh class | |  |  |  |  |
|  | A/B | 155 | 137 | 88.4 | P = 0.347 |
|  | C | 45 | 36 | 80.0 |  |
|  | Unknown | 76 | 66 | 86.8 |  |

TABLE S6 Specificities of the DT-HBT for healthy individuals.

|  | | N | Negative results (N) | Specificity (%) | Chi-square test, P value |
| --- | --- | --- | --- | --- | --- |
| Total | | 258 | 256 | 99.22 |  |
| Age | |  |  |  |  |
|  | ≤ 49 | 118 | 117 | 99.2 | P = 1.000 |
|  | 50-59 | 71 | 70 | 98.6 |  |
|  | 60-69 | 45 | 45 | 100 |  |
|  | ≥ 70 | 24 | 24 | 100 |  |
| Sex | |  |  |  |  |
|  | Male | 104 | 104 | 100 | P = 0.571 |
|  | Female | 154 | 152 | 98.7 |  |

**
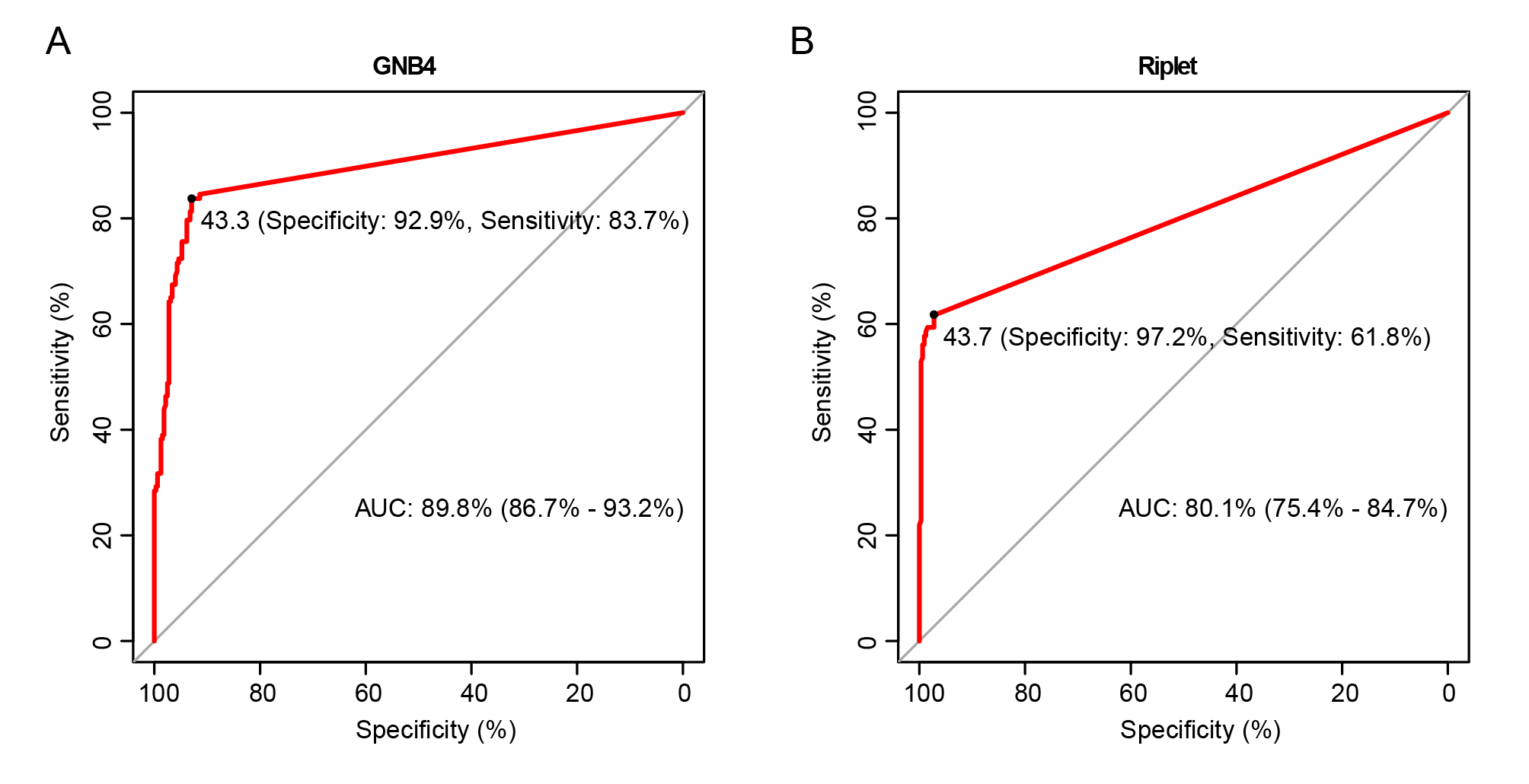
**

FIGURE S1 ROC analysis was performed on the Ct value of GNB4 and Riplet to determine the cut-off value of the DT-HBT. A: The Ct values of GNB4 were analyzed by ROC analysis in training set; B: The Ct values of Riplet were analyzed by ROC analysis in training set. The black dot represents the point where the Youden index is maximum, the Ct value corresponding to which is the threshold for positive determination of the target gene. In the training set, there were a total of 449 samples, including 123 HCC cases and 326 non-HCC cases.
